# The predictive value of preoperative inflammatory status for anastomotic leakage after esophagectomy for esophageal cancer

**DOI:** 10.3389/fonc.2025.1587586

**Published:** 2025-08-06

**Authors:** Zhulin Wang, Biao Wang, Dengguo Zhang, Chunyao Huang, Shaowu Sun, Kaiyuan Li, Yu Yi, Guoqing Zhang, Xiangnan Li, Jiangtao Pu

**Affiliations:** ^1^ Department of Thoracic Surgery, Affiliated Hospital of Southwest Medical University, Luzhou, Sichuan, China; ^2^ Department of Thoracic Surgery, First Affiliated Hospital of Zhengzhou University, Zhengzhou, Henan, China; ^3^ Operating Room of the Affiliated Hospital of Southwest Medical University, Luzhou, Sichuan, China

**Keywords:** anastomotic leakage, esophagectomy, inflammatory status, nomogram model, McKeown procedures

## Abstract

**Background:**

Anastomotic leakage is one of the most severe complications after esophageal cancer surgery. The purpose of this study was to evaluate the impact of preoperative inflammatory status on anastomotic leakage after esophageal cancer surgery and to construct a model for predicting anastomotic leakage after esophageal cancer surgery.

**Methods:**

A retrospective analysis was conducted on 1106 patients with esophageal cancer who underwent surgical treatment between September 2018 and December 2022. Patients were randomly divided into training and testing sets at a ratio of 7:3. Logistic regression analysis and LASSO regression analysis were performed on the training set. Independent influencing factors selected from the analysis were used for model construction. Internal validation was then performed.

**Results:**

A total of 1106 patients with esophageal cancer, with a mean age of 64.05 years, were included in our study. Among them, there were 785 male patients (71.0%) and 321 female patients (29.0%). Multivariate analysis revealed that a history of smoking (OR = 2.121, P = 0.016; 95% CI, 1.151-3.938), history of diabetes mellitus (OR = 5.473, P < 0.001; 95% CI, 2.587-11.382), high NMR (OR = 3.423, P = 0.002; 95% CI, 1.628-7.489), high PLR (OR = 3.675, P < 0.002; 95% CI, 1.642-8.406), and low PLT (OR = 0.986, P = < 0.001; 95% CI, 0.980-0.993) were independent risk factors for anastomotic leakage after esophageal cancer surgery. A forest plot was constructed for the independent risk factors, and the ROC curve analysis results showed that the model had good predictive ability in both the training and testing sets. Additionally, calibration curve and DCA curve analyses showed that the model had good predictive ability and net benefit.

**Conclusion:**

This study found that smoking history, diabetes history and preoperative inflammatory status (preoperative high NMR, high PLR, and low PLT) were risk factors for postoperative anastomotic leakage in patients with esophageal cancer. Based on these findings, we constructed a model for predicting anastomotic leakage after esophageal cancer surgery that demonstrated good predictive ability.

## Introduction

Esophageal cancer is the eighth most common cancer worldwide and the sixth leading cause of cancer-related deaths globally (1, 2). With the advancement of minimally invasive surgery, esophageal cancer surgery has also entered the era of minimally invasive techniques, primarily including the thoracoscopic and laparoscopic combined McKeown procedures and minimally invasive Ivor Lewis surgery, among others. Despite strengthened perioperative management in recent years, postoperative complications of esophageal cancer surgery still occur occasionally. The occurrence of postoperative complications significantly increases the economic burden on patients and leads to adverse prognoses.

Anastomotic leakage is one of the most serious complications following esophageal cancer surgery. It increases the length of hospital stay and hospitalization costs, imposing a severe financial burden on patients’ families. Additionally, anastomotic leakage significantly increases the risk of perioperative mortality. Therefore, many previous studies have focused on exploring the mechanisms behind the occurrence of anastomotic leaks and how to reduce their incidence. For example, the occurrence of anastomotic leaks may be related to factors such as blood supply (3, 4) and tension (5) at the anastomosis site. However, it is currently unclear whether this finding is related to the patient’s preoperative systemic inflammatory response status, among other factors. At present, there is no simple or effective method for predicting the risk of anastomotic leak occurrence.

Previous studies (6–9) have indicated that preoperative inflammatory markers (which are calculated based on various preoperative laboratory indicators) can effectively predict the prognosis of esophageal cancer. However, there are no studies on the predictive role of inflammatory markers for anastomotic leaks in esophageal cancer patients. Therefore, this study retrospectively analyzed the clinical data of 1,106 esophageal cancer patients treated with the McKeown procedure in our department of thoracic surgery from September 2018 to December 2022 to explore the predictive value of inflammatory markers for the occurrence of anastomotic leaks after esophageal cancer surgery.

## Materials and methods

### Patients

This study included 1,106 patients with esophageal cancer treated at the Department of Thoracic Surgery at the First Affiliated Hospital of Zhengzhou University from September 2018 to December 2022. We recorded the following preoperative clinical data: age, sex, neoadjuvant therapy, comorbidities, histological type, TNM stage, tumor location, tumor size, cardiopulmonary function, and preoperative laboratory indicators (Hb, WBC, Neut, Mono, ALB, PLT, etc.). Additionally, we calculated the following inflammatory markers based on the preoperative blood cell count: NLR (neutrophil-to-lymphocyte ratio), PLR (platelet-to-lymphocyte ratio), NMR (neutrophil-to-monocyte ratio), LMR (lymphocyte-to-monocyte ratio), SII (systemic immune-inflammation index = platelets × neutrophils/lymphocytes), NLPR (neutrophil-to-(lymphocyte × platelet) ratio), SIRI (systemic inflammation response index = neutrophils × monocytes/lymphocytes), AISI (aggregate inflammation score index = neutrophils × platelets × monocytes/lymphocytes), and MSIS (modified systemic inflammation score: a) ALB ≥40 g/L and LMR ≥3.4 were assigned a score of 0, b) either ALB <40 g/L or LMR < 3.4 were assigned a score of 1, and c) ALB <40 g/L and LMR < 3.4 were assigned a score of 2), and PNI (prognostic nutritional index = serum albumin + 5 × total lymphocyte count). Using ROC curves, we calculated the maximum sensitivity and specificity of these inflammatory markers, selected the cutoff values for the parameters, and classified patients into high-level and low-level groups based on each cutoff value.

### Inclusion and exclusion criteria

Inclusion Criteria:

1. Patients diagnosed with primary esophageal cancer through preoperative gastroscopy and pathological examination.
2. Preoperative computed tomography (CT) scans had been performed, with laboratory tests conducted within one week prior to surgery, including a complete blood count; liver and kidney function tests; electrolyte, blood biochemistry, and coagulation profile analyses; urinalysis; and fecal analysis.
3. Patients who underwent minimally invasive McKeown esophagectomy.
4. Patients with complete hospital records.
5. Patients with more than 3 months of comprehensive postoperative follow-up data.

Exclusion Criteria:

1. Patients with missing preoperative laboratory indicators.
2. Patients who required conversion to open thoracotomy during surgery.
3. Patients who underwent concurrent laryngectomy.
4. Patients with missing postoperative follow-up data.

**Figure 1:**
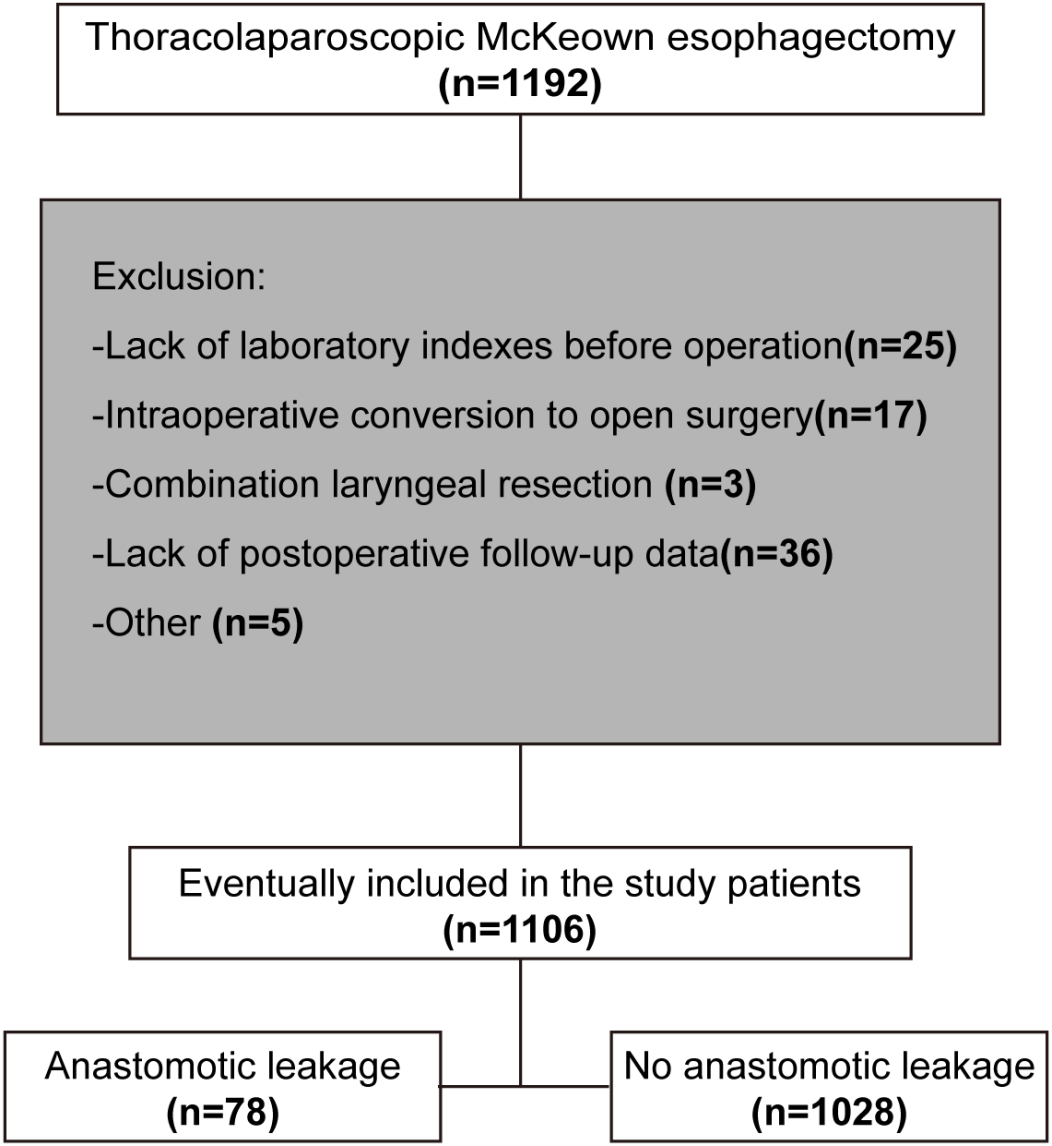
Patient screening flow chart.

The screening process is illustrated in **Figure 1**.

### Management of patients undergoing esophageal cancer surgery

All patients were confirmed to have primary esophageal cancer through preoperative gastroscopy and pathological examination. Additionally, all patients underwent preoperative thoracoabdominal enhanced CT, cranial MRI, and neck ultrasonography to exclude distant metastasis. Patients who were found to have distant metastases were excluded from surgical treatment. Cardiopulmonary function tests were conducted to assess whether patients could tolerate the surgery. Laboratory tests (including a complete blood count; liver and kidney function tests; electrolyte, blood biochemistry, and coagulation profile analyses; urinalysis; and stool analysis) were performed within one week before surgery.

Surgical Procedure: All patients underwent the McKeown procedure with thoracoscopic assistance (right thoracic, left cervical, and abdominal incisions) and esophageal reconstruction using a mechanical stapler for end-to-side anastomosis. All surgeries adhered to the principles of radical esophagectomy: the margin of the esophageal tumor remnant was more than 5 cm, and lymph node dissection was performed simultaneously.

All patients had a chest CT scan one week postoperatively. In addition, patients who developed unexplained fever or had phlegm-like drainage from the neck or chest tubes underwent gastrointestinal contrast studies or endoscopy to determine whether an esophageal anastomotic leak had occurred. All patients were followed up with a chest CT scan at the outpatient clinic one month after surgery, and patients suspected of having an anastomotic leak underwent a contrast swallow study or endoscopy for verification.

### Definition of esophageal anastomotic leak in this study

The primary outcome measure in this study was anastomotic leakage, defined as the extravasation of water-soluble contrast agent during a contrast swallow study or on CT scan, visible separation or fistula at the anastomosis during endoscopic examination, or the presence of saliva leakage through the cervical wound (3). Postoperative bile or phlegm discharge through the chest tube was also considered (10).

### Statistical methods

We performed the statistical analysis using the R language. Categorical data are expressed as frequencies, and continuous data are represented as the mean ± standard deviation. Chi-square tests and t tests were used for statistical analysis. Patients were randomly divided into a training set and a test set at a 7:3 ratio. Univariate logistic regression analysis was conducted on the training set, and a P value <0.05 was considered a potential risk factor for postoperative esophageal anastomotic leakage. Factors identified by univariate logistic regression analysis were further analyzed using LASSO regression. The optimal lambda (Lambda.1se), which is the largest lambda within one standard deviation of the minimum error, was chosen for the best lambda. The selected factors were then analyzed using multivariate logistic regression. Factors with a P value <0.05 in the multivariate logistic regression analysis were considered independent risk factors for postoperative esophageal anastomotic leakage, and these factors were used to construct the model. Nomograms were drawn to predict the incidence of postoperative esophageal anastomotic leaks and were internally validated. The discrimination and calibration abilities of the model were evaluated using ROC curve and calibration curve analyses, while the predictive ability and net benefit of the model were assessed using decision curve analysis (DCA).

## Results

### Cutoff values for inflammatory markers

Using ROC curve analysis, the maximum sensitivity and specificity of these inflammatory markers were calculated to determine the optimal thresholds for predicting esophageal anastomotic leaks, as shown in **Supplementary Figure 1**. The best cutoff values for the inflammatory markers NLPR, AISI, SIRI, NMR, PNI, NLR, LMR, PLR, and SII were 0.010, 113.923, 1.130, 7.753, 50.625, 2.331, 4.303, 151.573, and 392.944, respectively. The corresponding sensitivities and specificities are presented in **Table 1**. According to the cutoff values of the inflammatory markers, 1,106 patients were divided into two groups.

**Table 1:** Diagnostic value of the parameters.

Parameters	Cutoff value	Sensitivity	Specificity	AUC	95% CI	*P* value
NLPR	0.010	0.756	0.567	0.724	0.667-0.780	<0.001
AISI	113.923	0.821	0.331	0.566	0.500-0.629	0.057
SIRI	1.130	0.449	0.732	0.622	0.559-0.685	<0.001
NMR	7.753	0.654	0.538	0.608	0.544-0.685	0.001
mSIS	-	0.782	0.389	0.582	0.521-0.643	0.015
PNI	50.625	0.769	0.456	0.633	0.569-0.696	<0.001
NLR	2.331	0.577	0.713	0.697	0.638-0.756	<0.001
LMR	4.303	0.769	0.446	0.627	0.563-0.691	<0.001
PLR	151.573	0.462	0.725	0.586	0.517-0.656	0.011
SII	392.944	0.667	0.503	0.606	0.541-0.670	0.002

### Patients

In our study, a total of 1,106 patients with esophageal cancer were included. The demographic and baseline data of the patients in the study cohort, with an average age of 64.05 years, are shown in **Table 2**. There were 785 male patients (71.0%) and 321 female patients (29.0%). The cohort predominantly consisted of esophageal cancer patients with squamous cell carcinoma of the middle and lower esophagus, stage I–II, and without adjuvant therapy. The average tumor diameter was 3.19 cm, and the average surgery duration was 312.36 minutes. Additionally, the included patients were randomly divided into a training set (n=774) and a validation set (n=332) at a 7:3 ratio, with no statistically significant differences in demographic or clinical characteristics between the training and validation cohorts (p>0.05) (**Supplementary Table 1**).

**Table 2:** Demographic and baseline data of the study cohort.

Characteristic	AL (n=78)	none-AL (n=1028)	All	p
Age	63.96±8.517	64.06±7.602	64.05±7.666	0.914
Sex				0.048
Male	63(80.8%)	722(70.2%)	785(71.0%)	
Female	15(19.2%)	306(29.8%)	321(29.0%)	
BMI	23.43±3.11	23.91±3.369	23.88±3.353	0.219
Smoking				0.002
No	36(46.2%)	652(63.4%)	688(62.2%)	
Yes	42(53.8%)	376(36.6%)	418(37.8%)	
Drinking				0.512
No	57(73.1%)	785(76.4%)	842(76.1%)	
Yes	21(26.9%)	243(23.6%)	264(23.9%)	
History of lung disease				0.340
No	71(91.0%)	964(93.6%)	1035(93.6%)	
Yes	7(9.0%)	64(6.2%)	71(6.4%)	
Diabetes				<0.001
No	49(62.8%)	949(92.3%)	998(90.2%)	
Yes	29(37.2%)	79(7.7%)	108(9.8%)	
Hypertension				0.808
No	58(74.4%)	777(75.6%)	835(75.5%)	
Yes	20(25.6%)	251(24.4%)	271(24.5%)	
Coronary heart disease				0.079
No	70(89.7%)	972(94.6%)	1042(94.2%)	
Yes	8(10.3%)	56(5.4%)	62(5.8%)	
Surgical history				0.161
No	56(71.8%)	808(78.6%)	864(78.1%)	
Yes	22(28.2%)	220(21.4%)	242(21.9%)	
Neoadjuvant therapy				0.716
No	59(75.6%)	796(77.4%)	855(77.3%)	
Yes	19(24.4%)	232(22.6%)	251(22.7%)	
Tumor location				0.940
Upper	11(14.1%)	139(13.5%)	150(13.6%)	
Middle	27(34.6%)	344(33.5%)	371(33.5%)	
Lower	32(41.0%)	455(44.3%)	487(44.0%)	
GEJ	8(10.3%)	90(8.8%)	98(8.9%)	
Histological type				0.263
Squamous	63(80.8%)	853(83.0%)	916(82.8%)	
Adenocarcinoma	7(9.0%)	115(11.2%)	122(11.0%)	
Other	8(10.3%)	60(5.8%)	68(6.1%)	
T				0.716
T1	25(32.1%)	393(38.2%)	418(37.8%)	
T2	20(25.6%)	254(24.7%)	274(24.8%)	
T3	32(41.0%)	372 (36.2%)	404(36.5%)	
T4	1 (1.3%)	9 (0.9%)	10(0.9%)	
N				0.816
N0	49(62.8%)	629(61.2%)	678(61.3%)	
N1	14(17.9%)	226 (22.0%)	240(21.7%)	
N2	12 (15.4%)	132 (12.8%)	144(13.0%)	
N3	3 (3.8%)	41 (4.0%)	44(4.0%)	
TNM				0.937
1	22(28.2%)	319(31.0%)	341(30.8%)	
2	29 (37.2%)	374 (36.4%)	403(36.4%)	
3	24 (30.8%)	290 (28.2%)	314(28.4%)	
4	3 (3.8%)	45 (4.4%)	48(4.3%)	
NLPR				<0.001
<0.010	20(25.6%)	585(56.9%)	605(54.7%)	
≥0.010	58(74.4%)	443(43.1%)	501(45.3%)	
AISI				0.006
<113.923	14(17.9%)	340(33.1%)	354(32.0%)	
≥113.923	64 (82.1%)	688 (66.9%)	752(68.0%)	
SIRI				0.001
<1.130	43(55.1%)	753(73.2%)	796(72.0%)	
≥1.130	35 (44.9%)	275 (26.8%)	310(28.0%)	
NMR				<0.001
<7.753	25(32.1%)	553(53.8%)	578(52.3%)	
≥7.753	53(67.9%)	475(46.2%)	528(47.7%)	
MSIS				0.011
0	17(21.8%)	400(38.9%)	417(37.7%)	
1	43(55.1%)	434 (42.2%)	477(43.1%)	
2	18 (23.1%)	194 (18.9%)	212(19.2%)	
PNI				<0.001
<50.625	60(76.9%)	559(54.4%)	619(56.0%)	
≥50.625	18(23.1%)	469(45.6%)	487(44.0%)	
NLR				<0.001
<2.331	33(42.3%)	733(71.3%)	766(69.3%)	
≥2.331	45(57.7%)	295(28.7%)	340(30.7%)	
LMR				<0.001
<4.303	60(76.9%)	570(55.4%)	630(57.0%)	
≥4.303	18(23.1%)	458(44.6%)	476(43.0%)	
PLR				<0.001
<151.573	40(51.3%)	745(72.5%)	785(71.0%)	
≥151.573	38(48.7%)	283(27.5%)	321(29.0%)	
SII				0.004
<392.944	26(33.3%)	517(50.3%)	543(49.1%)	
≥392.944	52(66.7%)	511(49.7%)	563(50.9%)	
WBC	5.73±1.977	5.73±1.817	5.74±1.827	0.990
RBC	4.16±0.537	4.19±0.570	4.20±0.568	0.587
Hb	129.24±14.868	129.48±16.414	129.05±16.304	0.900
PLT	190.55±60.801	213.68±61.902	212.05±62.081	0.001
Neut	3.74±1.694	3.38±1.467	3.40±1.486	0.038
Lymp	1.43±0.726	1.78±1.164	1.75±1.142	0.010
Mono	0.49±0.082	0.48±0.017	0.48±0.036	0.899
ALB	40.89±3.491	41.54±3.671	41.49±3.661	0.131
Prealb	220.73±44.569	226.11±50.041	225.73±49.676	0.357
PT	10.549±1.025	10.48±0.842	10.49±0.856	0.519
INR	0.95±0.124	1.57±0.239	1.53±0.236	0.475
APTT	28.95±3.438	29.09±3.573	29.08±3.562	0.749
TT	15.24±2.952	15.49±2.016	15.474±2.095	0.298
FVC	3.56±0.825	3.57±1.477	3.57±1.440	0.935
FEV1	2.65±0.679	2.63±0.639	2.63±0.642	0.750
FEV%	74.42±10.074	75.42±23.73	75.38±23.036	0.820
DLCO	6.87±1.719	7.17±1.727	7.15±1.727	0.135
EF	63.42±1.798	63.38±2.160	63.38±2.136	0.853
Tumor size	3.34±1.558	3.18±1.497	3.19±1.501	0.381
Operation time	321.14±57.955	311.69±47.589	312.36±48.423	0.097
Intraoperative infusion	3334.62±491.176	3294.25±561.403	3319.54±816.153	0.537

BMI, body mass index; GEJ, gastro-oesophageal junction cancers; FVC, forced vital capacity; FEV1, forced expiratory volume in one second; DLCO, Diffusing capacity of the lung for carbon monoxide; EF, ejection fraction.

### Screening for predictive factors of anastomotic leakage after esophageal cancer surgery

**Figure 2:**
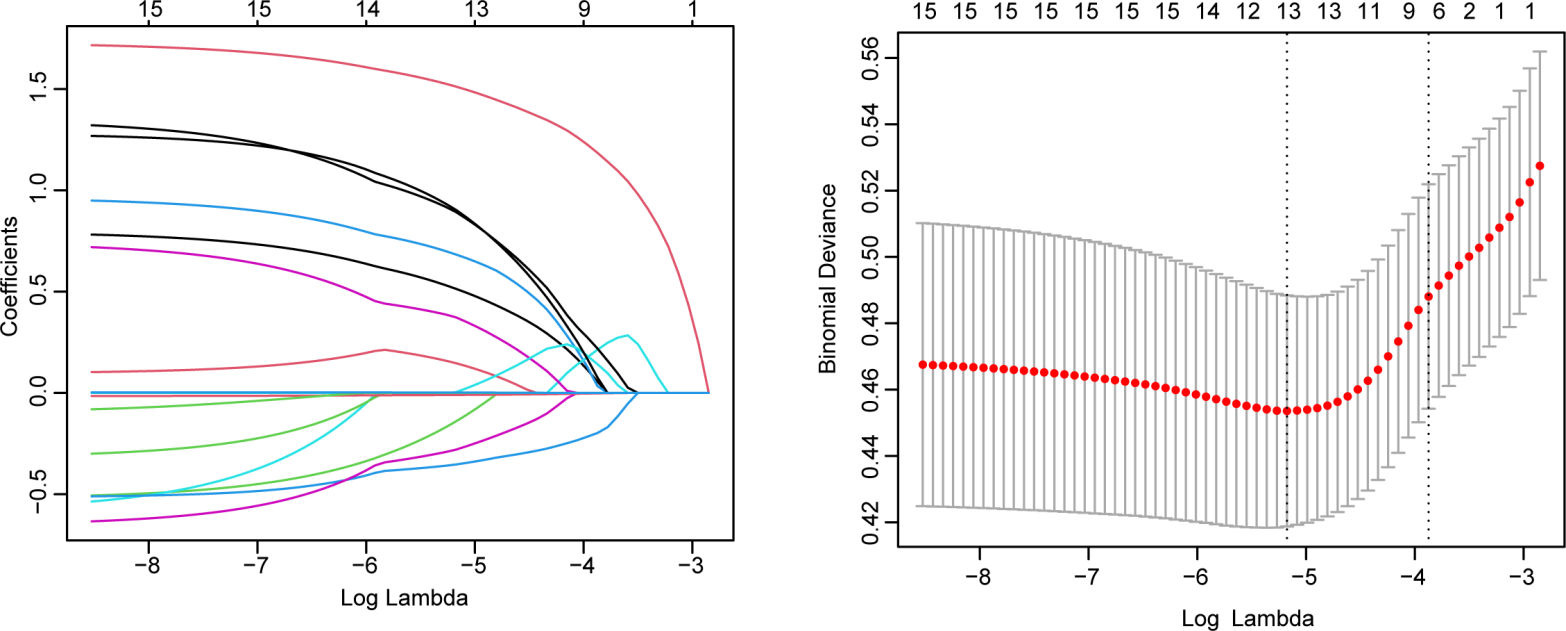
LASSO regression analysis for selecting influencing factors of anastomotic leakage after esophageal cancer surgery.

In the training set, univariate logistic regression analysis identified 14 potential influencing factors (**Supplementary Table 2**). These 14 factors were then included in a LASSO regression for further analysis, from which 12 factors were selected for inclusion in multivariate logistic regression (**Figure 2**). Ultimately, in the multivariate analysis, having a history of smoking (OR = 2.121, P = 0.016; 95% CI: 1.151-3.938), history of diabetes (OR = 5.473, P < 0.001; 95% CI: 2.587-11.382), high NMR (OR = 3.423, P = 0.002; 95% CI: 1.628-7.489), high PLR (OR = 3.675, P =0.002; 95% CI: 1.642-8.406), and low PLT (OR = 0.986, P <0.001; 95% CI: 0.980-0.993) were found to be independent risk factors for anastomotic leakage after esophageal cancer surgery (**Table 3**).

**Table 3:** Multivariate logistic regression analysis.

Characteristic	Multivariable		
	OR	95%CI	P
Smoking			
No			
Yes	2.121	1.151-3.938	0.016
Diabetes			
No			
Yes	5.473	2.587-11.382	<0.001
Histological type			
Squamous			
Adenocarcinoma	0.58	0.179-1.532	0.312
Other	2.348	0.818-6.033	0.091
SIRI			
<1.130			
≥1.130	1.945	0.834-4.586	0.125
NMR			
<7.753			
≥7.753	3.423	1.628-7.489	0.002
MSIS			
0			
1	1.118	0.447-2.868	0.813
2	0.715	0.211-2.476	0.593
PNI			
<50.625			
≥50.625	0.586	0.255-1.296	0.194
NLR			
<2.331			
≥2.331	0.813	0.328-1.989	0.652
LMR			
<4.303			
≥4.303	0.561	0.221-1.395	0.216
PLR			
<151.573			
≥151.573	3.675	1.642-8.406	0.002
PLT	0.986	0.980-0.993	<0.001
Time	1.003	0.997-1.008	0.253

### Construction of the nomogram model

**Figure 3:**
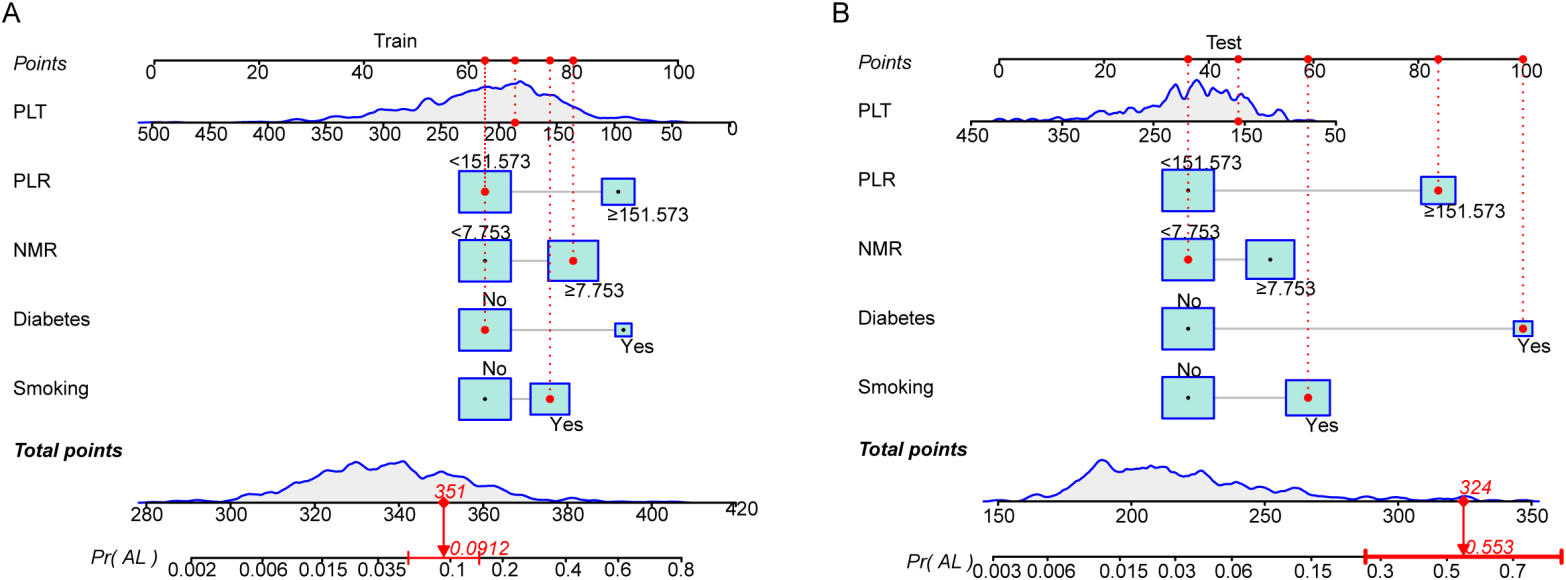
**(A)**, Nomogram of the predictive model in the training set; **(B)**, Nomogram of the predictive model in the test set.

We constructed a nomogram with the independent influencing factors identified by multivariate Cox regression analysis in the training set to predict the risk of anastomotic leakage after esophageal cancer surgery. Each influencing factor is assigned a corresponding point, allowing the risk contributed by each factor to be converted into a calculable value. The total score is calculated by summing the scores of all influencing factors, and based on this total score, the corresponding probability of anastomotic leak occurrence can be determined (**Figure 3A** and **Supplementary Figure 2**). For example, in the diagram, we show a patient with a total score of 351, corresponding to a 9.12% probability of experiencing an anastomotic leak. Furthermore, using the parameters of this model, we plotted a corresponding nomogram for the validation set (**Figure 3B**).

### Evaluation and validation of the nomogram model

**Figure 4:**
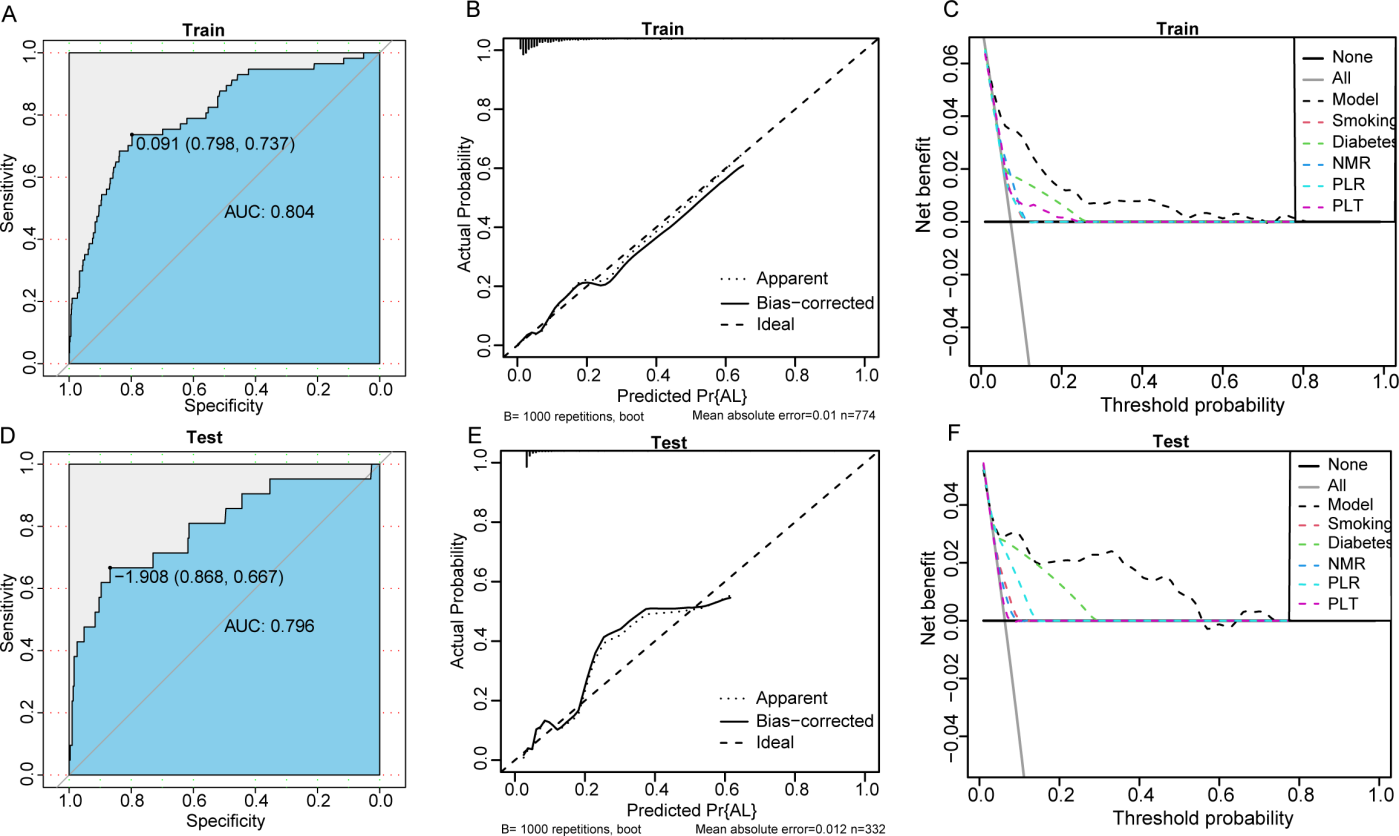
**(A–C)**, ROC curves, calibration curves, and DCA curves in the training set; **(D–F)**, ROC curves, calibration curves, and DCA curves in the test set.

We assessed the effectiveness and clinical applicability of the model using ROC curve analysis. In the training set, the AUC value was 0.804, with an optimal cutoff value of 0.091, at which the sensitivity and specificity of the model was 73.7% and 79.8%, respectively. In the test set, the AUC value of the model was 0.796, with an optimal cutoff value of -1.908, at which the sensitivity and specificity of the model was 66.7% and 86.8%, respectively. In the training and test sets, the calibration curves indicated good agreement between the predicted occurrences of anastomotic leaks and the actual occurrence rates. The decision curve analysis (DCA) results demonstrated that, in both the training and test sets, this model provided better net benefit and clinical effectiveness than did the other independent influencing factors (**Figure 4**).

## Discussion

In this study, 1,106 patients who underwent thoracoscopic and laparoscopic McKeown esophagectomy for esophageal cancer were analyzed to investigate whether inflammatory markers such as the NLPR, AISI, mSIS, SIRI, NMR, PNI, NLR, LMR, PLR, and SII could serve as predictive tools for anastomotic leaks after esophageal cancer surgery. We found that a history of smoking, history of diabetes, high NMR, high PLR, and low PLT were independent risk factors for anastomotic leaks after esophageal cancer surgery. Moreover, we constructed a nomogram that accurately predicted the risk of anastomotic leaks in patients after esophageal cancer surgery, and it showed favorable results in internal validation.

The nomogram model quantifies patient risk scores by integrating multiple clinical indicators to predict the probability of postoperative anastomotic leakage in esophageal cancer patients. This model facilitates preoperative risk stratification of patients, enabling medical teams to implement targeted preventive measures and reduce anastomotic leakage incidence. Moreover, in combination with specific patient risk prediction results, the nomogram model can aid surgical teams in creating more precise surgical plans. For instance, for patients predicted to have a higher possibility of postoperative anastomotic leakage, the surgical team might opt for a more conservative surgical approach to minimize potential risks. Such individualized treatment plans can better accommodate individual patient characteristics, thereby enhancing surgical success rates and safety.

Previous studies have shown that neutrophils are capable of releasing a variety of substances that can destroy cells and dissolve connective tissues (11). Neutrophils can mediate tissue-destructive events, even in the absence of pathogens. Neutrophils are also capable of releasing certain cytokines, most notably IL1 and TNFα, which may contribute to tissue damage under certain conditions. Neutrophil granules contain a variety of enzymes capable of degrading nearly all components of the extracellular matrix and cleaving several key plasma proteins (12). In preclinical models (13), neutrophils also promoted atherosclerosis in a stage-dependent manner. Activated neutrophils undergo degranulation, which enhances monocyte recruitment, and secrete reactive oxygen species (ROS) and proteases, leading to endothelial dysfunction. This allows the extravasation of LDL cholesterol, further contributing to the development of atherosclerosis. During an inflammatory response, neutrophils traverse microvascular walls, which serve as a primary barrier, to exert their lytic functions. This leads to the excessive flow of blood components and fluid into the surrounding tissues, causing local tissue edema (14). Therefore, neutrophils may cause anastomotic leaks through direct damage to the local tissues of the esophageal anastomosis and local vascular injury.

Monocytes are blood-derived mononuclear phagocytes that are distributed throughout the body. Monocytes in circulation leave the bloodstream and migrate into tissues, where they differentiate into macrophages or dendritic cells under the regulation of local growth factors, proinflammatory cytokines, and microbial products (15). Monocytes are a crucial component of the mononuclear phagocyte system (MPS) and play a significant role in many inflammatory diseases, such as infections, cardiovascular diseases, type I diabetes, and cancer (16). Furthermore, monocytes play a crucial role in vascular repair and remodeling by secreting a variety of cytokines, growth factors, and extracellular matrix (ECM)-remodeling enzymes (17).

Platelets help prevent blood loss at sites of vascular injury; can express and release substances that promote tissue repair; and influence processes such as angiogenesis, inflammation, and immune responses (18). Platelets are capable of releasing numerous proteins that are beneficial for wound healing and promoting angiogenesis. These include PDGF A, B, and C; insulin-like growth factor-1 (IGF-1); VEGF (essentially VEGF-A); connective tissue growth factor (CTGF); and various chemokines and cytokines (19). Platelet-associated tissue factor not only promotes thrombin generation but also may facilitate wound healing through direct induction of cultured smooth muscle cell migration, among other mechanisms (20). The release of growth factors, cytokines, or chemokines by platelets can regulate the release of matrix metalloproteinases (MMPs), a type of protease that promotes angiogenesis (21). Furthermore, previous studies (22) have shown that platelet-derived serotonin can also mediate liver regeneration. In our study, a lower preoperative platelet count was identified as an independent risk factor for the occurrence of esophageal anastomotic leaks, which may be related to the role of platelets in tissue repair.

Previous studies (23, 24) have shown that lymphocytes, especially regulatory T cells (Tregs), can promote the repair and regeneration of various tissues. In many tissues, Tregs are recruited to injury sites to accelerate the resolution of inflammation and regulate immunity postinjury (25). Tregs have a wide range of regenerative effects, such as promoting the restoration of blood flow after ischemia, controlling adipose tissue inflammation, enhancing muscle repair, and maintaining tissue/organ homeostasis (26). Tregs can increase the differentiation of stem/progenitor cells such as satellite cells to replace damaged skeletal muscle and increase the proliferation of newly formed cardiomyocytes for functional regeneration (25). Additionally, certain subsets of T lymphocytes can stimulate wound healing under normal conditions (27). When T lymphocytes are depleted using specific monoclonal antibodies (Mabs), wound healing is impaired, as manifested by reduced wound tensile strength and collagen synthesis (28). In addition to T lymphocytes, NK cells and CD4 T cells have also been shown to regulate arteriogenesis in mouse ischemia models (29).

Furthermore, our study revealed that a history of smoking and a history of diabetes were independent risk factors for the occurrence of esophageal anastomotic leaks, which is consistent with the findings of several previous studies (30–34).

This study has several limitations. Although internal validation was conducted, this is a single-center retrospective study without external data validation, which may lead to insufficient sample heterogeneity and an inability to fully represent a broader population. A single-center retrospective study is unable to strictly control for confounding factors, and there may be potential confounding factors that are difficult to identify and control. These confounding factors may mask or exaggerate the true effects of the study results, leading to biases. Further prospective studies are needed to better control for potential confounding factors stemming from patient characteristics. Additionally, the causes of anastomotic leaks following esophageal cancer surgery vary. This study focused on analyzing the preoperative systemic inflammatory status of patients with esophageal cancer and did not involve an analysis of factors such as tension at the anastomosis site or blood supply.

## Conclusion

In our study, a history of smoking, history of diabetes, high preoperative NMR, high PLR, and low PLT were identified as independent risk factors for anastomotic leaks after esophageal cancer surgery. Based on these findings, we constructed a model to predict anastomotic leaks after esophageal cancer surgery. The evaluation of the model using ROC curve analysis showed that it has good predictive ability in both the training and test sets. Furthermore, calibration curve analysis and decision curve analysis (DCA) indicated that the model has good predictive performance and net benefit.

## Data Availability

The raw data supporting the conclusions of this article will be made available by the authors, without undue reservation.

## Supplementary material

Supplementary Figure 1 Utilizing ROC curve analysis to determine the optimal cutoff values for inflammatory markers.

Supplementary Figure 2 A high-resolution nomogram of the training set.
