# Supplementary material for: The predictive value of preoperative inflammatory status for anastomotic leakage after esophagectomy for esophageal cancer
Source: Front Oncol. 2025 Aug 6;15:1587586. doi: 10.3389/fonc.2025.1587586 (PMC12364653; doi:10.3389/fonc.2025.1587586)
Supplement: Supplementary file 1 [file Table1.docx]

Supplementary Table 1, Demographic and clinical characteristics of the training and validation groups

| Characteristic | Train(n=774) | Test(n=332) | All | p |
| --- | --- | --- | --- | --- |
| Age | 63.93±7.675 | 64.33±7.647 | 64.05±7.666 | 0.423 |
| Sex |  |  |  | 0.288 |
| Male | 542(70.0%) | 243(73.2%) | 785(71.0%) |  |
| Female | 232(30.0%) | 89(26.8%) | 321(29.0%) |  |
| BMI | 23.92±3.281 | 23.76±3.516 | 23.88±3.353 | 0.464 |
| Smoking |  |  |  | 0.067 |
| No | 495(64.0%) | 193(58.1%) | 688(62.2%) |  |
| Yes | 279(36.0%) | 139(41.9%) | 418(37.8%) |  |
| Drinking |  |  |  | 0.299 |
| No | 596(77.0%) | 246(74.1%) | 842(76.1%) |  |
| Yes | 178(23.0%) | 86(25.9%) | 264(23.9%) |  |
| History of lung disease |  |  |  | 0.652 |
| No | 726(93.8%) | 309(93.1%) | 1035(93.6%) |  |
| Yes | 48(6.2%) | 23(6.9%) | 71(6.4%) |  |
| Diabetes |  |  |  | 0.217 |
| No | 704(91.0%) | 294(88.6%) | 998(90.2%) |  |
| Yes | 70(9.0%) | 38(11.4%) | 108(9.8%) |  |
| Hypertension |  |  |  | 0.578 |
| No | 588(76.0%) | 247(74.4%) | 835(75.5%) |  |
| Yes | 186(24.0%) | 85(25.6%) | 271(24.5%) |  |
| Coronary heart disease |  |  |  | 0.367 |
| No | 726(93.8%) | 316(95.2%) | 1042(94.2%) |  |
| Yes | 48(6.2%) | 16(4.8%) | 62(5.8%) |  |
| Surgical history |  |  |  | 0.243 |
| No | 612(79.1%) | 252(75.9%) | 864(78.1%) |  |
| Yes | 162(20.9%) | 80(24.1%) | 242(21.9%) |  |
| Neoadjuvant therapy |  |  |  | 0.143 |
| No | 589(76.1%) | 266(80.1%) | 855(77.3%) |  |
| Yes | 185(23.9%) | 66(19.9%) | 251(22.7%) |  |
| Tumor location |  |  |  | 0.568 |
| Upper | 103(13.3%) | 47(14.2%) | 150(13.6%) |  |
| Middle | 251(32.4%) | 120(36.1%) | 371(33.5%) |  |
| Lower | 349(45.1%) | 138(41.6%) | 487(44.0%) |  |
| GEJ | 71(9.2%) | 27(8.1%) | 98(8.9%) |  |
| Histological type |  |  |  | 0.118 |
| Squamous | 642(82.9%) | 274(82.5%) | 916(82.8%) |  |
| Adenocarcinoma | 91(11.8%) | 31(9.3%) | 122(11.0%) |  |
| Other | 41(5.3%) | 27(8.1%) | 68(6.1%) |  |
| T |  |  |  | 0.522 |
| T1 | 283(36.6%) | 135(40.7%) | 418(37.8%) |  |
| T2 | 198(25.6%) | 76(22.9%) | 274(24.8%) |  |
| T3 | 285(36.8%) | 119(35.8%) | 404(36.5%) |  |
| T4 | 8 (1.0%) | 2 (0.6%) | 10(0.9%) |  |
| N |  |  |  | 0.404 |
| N0 | 465(60.1%) | 213(64.2%) | 678(61.3%) |  |
| N1 | 172 (22.2%) | 68 (20.5%) | 240(21.7%) |  |
| N2 | 102 (13.2%) | 42 (12.7%) | 144(13.0%) |  |
| N3 | 35 (4.5%) | 9(2.7%) | 44(4.0%) |  |
| TNM |  |  |  | 0.140 |
| 1 | 223(30.1%) | 108(32.5%) | 341(30.8%) |  |
| 2 | 272 (35.1%) | 131 (39.5%) | 403(36.4%) |  |
| 3 | 231 (29.8%) | 83 (25.0%) | 314(28.4%) |  |
| 4 | 38(4.9%) | 10(3.0%) | 48(4.3%) |  |
| NLPR |  |  |  | 0.316 |
| <0.010 | 431(55.7%) | 174(52.4%) | 605(54.7%) |  |
| ≥0.010 | 343(44.3%) | 158(47.6%) | 501(45.3%) |  |
| AISI |  |  |  | 0.505 |
| <113.923 | 243(31.4%) | 111(33.4%) | 354(32.0%) |  |
| ≥113.923 | 531 (68.6%) | 332 (66.6%) | 752(68.0%) |  |
| SIRI |  |  |  | 0.385 |
| <1.130 | 563(72.7%) | 233(70.2%) | 796(72.0%) |  |
| ≥1.130 | 211 (27.3%) | 999 (29.8%) | 310(28.0%) |  |
| NMR |  |  |  | 0.555 |
| <7.753 | 40051.7%) | 178(53.6%) | 578(52.3%) |  |
| ≥7.753 | 374(48.3%) | 154(46.4%) | 528(47.7%) |  |
| MSIS |  |  |  | 0.166 |
| 0 | 278(35.9%) | 139(41.9%) | 417(37.7%) |  |
| 1 | 345(44.6%) | 132 (39.8%) | 477(43.1%) |  |
| 2 | 151 (19.5%) | 61 (18.4%) | 212(19.2%) |  |
| PNI |  |  |  | 0.875 |
| <50.625 | 432(55.8%) | 187(56.3%) | 619(56.0%) |  |
| ≥50.625 | 342(44.2%) | 145(43.7%) | 487(44.0%) |  |
| NLR |  |  |  | 0.993 |
| <2.331 | 536(69.3%) | 230(69.3%) | 766(69.3%) |  |
| ≥2.331 | 238(30.7%) | 102(30.7%) | 340(30.7%) |  |
| LMR |  |  |  | 0.498 |
| <4.303 | 446(57.6%) | 184(55.4%) | 630(57.0%) |  |
| ≥4.303 | 328(42.4%) | 148(44.6%) | 476(43.0%) |  |
| PLR |  |  |  | 0.502 |
| <151.573 | 554(71.6%) | 231(69.6%) | 785(71.0%) |  |
| ≥151.573 | 220(28.4%) | 101(30.4%) | 321(29.0%) |  |
| SII |  |  |  | 0.694 |
| <392.944 | 377(48.7%) | 166(50.0%) | 543(49.1%) |  |
| ≥392.944 | 397(51.3%) | 166(50.0%) | 563(50.9%) |  |
| WBC | 5.74±1.793 | 5.72±1.907 | 5.74±1.827 | 0.902 |
| RBC | 4.17±0.567 | 4.24±0.565 | 4.20±0.568 | 0.074 |
| Hb | 129.225±16.812 | 129.93±15.065 | 129.05±16.304 | 0.524 |
| PLT | 212.05±63.244 | 212.06±53.376 | 212.05±62.081 | 0.998 |
| Neut | 3.39±1.426 | 3.41±1.618 | 3.40±1.486 | 0.782 |
| Lymp | 1.77±1.314 | 1.71±1.314 | 1.75±1.142 | 0.441 |
| Mono | 0.48±0.018 | 0.51±0.038 | 0.48±0.036 | 0.490 |
| ALB | 41.44±3.727 | 41.61±3.505 | 41.49±3.661 | 0.488 |
| Prealb | 225.19±51.897 | 226.98±44.113 | 225.73±49.676 | 0.583 |
| PT | 10.479±0.885 | 10.51±0.785 | 10.49±0.856 | 0.544 |
| INR | 1.53±0.267 | 1.54±0.402 | 1.53±0.236 | 0.989 |
| APTT | 28.98±3.685 | 29.31±3.250 | 29.08±3.562 | 0.145 |
| TT | 15.45±2.172 | 15.52±1.907 | 15.474±2.095 | 0.603 |
| FVC | 3.59±1.645 | 3.52±0.780 | 3.57±1.440 | 0.438 |
| FEV1 | 2.64±0.644 | 2.63±0.636 | 2.63±0.642 | 0.942 |
| FEV% | 75.50±26.926 | 75.11±8.847 | 75.38±23.036 | 0.796 |
| DLCO | 7.16±1.762 | 7.14±1.645 | 7.15±1.727 | 0.859 |
| EF | 63.42±2.160 | 63.28±2.078 | 63.38±2.136 | 0.310 |
| Tumor size | 3.23±1.487 | 3.11±1.535 | 3.19±1.501 | 0.219 |
| Operation time | 312.60±48.594 | 311.79±48.091 | 312.36±48.423 | 0.798 |
| Intraoperative infusion | 3294.35±556.199 | 3303.49±558.441 | 3319.54±816.153 | 0.802 |
