# Supplementary material for: The predictive value of preoperative inflammatory status for anastomotic leakage after esophagectomy for esophageal cancer
Source: Front Oncol. 2025 Aug 6;15:1587586. doi: 10.3389/fonc.2025.1587586 (PMC12364653; doi:10.3389/fonc.2025.1587586)
Supplement: Supplementary file 2 [file Table2.docx]

Supplementary Table 2, Univariate logistic regression analysis of the training set

|  | Univariate | | |
| --- | --- | --- | --- |
| **Characteristic** | OR | 95%CI | P |
| Age | 1.023 | 0.987-1.060 | 0.217 |
| BMI | 0.936 | 0.857-1.021 | 0.137 |
| Sex |  |  |  |
| Male | 1 |  |  |
| Female | 0.745 | 0.399-1.391 | 0.356 |
| Smoking |  |  |  |
| No | 1 |  |  |
| Yes | 1.935 | 1.126-3.325 | 0.017* |
| Drinking |  |  |  |
| No | 1 |  |  |
| Yes | 1.214 | 0.656-2.245 | 0.537 |
| History of lung disease |  |  |  |
| No | 1 |  |  |
| Yes | 0.830 | 0.250-2.758 | 0.761 |
| Diabetes |  |  |  |
| No | 1 |  |  |
| Yes | 5.902 | 3.157-11.035 | <0.001* |
| Hypertension |  |  |  |
| No | 1 |  |  |
| Yes | 1.256 | 0.687-2.294 | 0.459 |
| Coronary heart disease |  |  |  |
| No | 1 |  |  |
| Yes | 2.308 | 0.985-5.409 | 0.054 |
| Surgical history |  |  |  |
| No | 1 |  |  |
| Yes | 1.385 | 0.747-2.566 | 0.301 |
| Neoadjuvant therapy |  |  |  |
| No | 1 |  |  |
| Yes | 1.149 | 0.622-2.124 | 0.657 |
| Tumor location |  |  |  |
| Upper | References |  |  |
| Middle | 0.973 | 0.412-2.298 | 0.949 |
| Lower | 0.916 | 0.400-2.098 | 0.836 |
| GEJ | 0.900 | 0.282-2.872 | 0.858 |
| Histological type |  |  |  |
| Squamous |  |  |  |
| Adenocarcinoma | 0.771 | 0.298-1.997 | 0.593 |
| Other | 2.731 | 1.146-6.507 | 0.023* |
| T |  |  |  |
| T1 | 1 |  |  |
| T2 | 1.467 | 0.715-3.008 | 0.296 |
| T3 | 1.534 | 0.797-2.955 | 0.200 |
| T4 | 2.384 | 0.276-20.572 | 0.429 |
| N |  |  |  |
| N0 | 1 |  |  |
| N1 | 1.015 | 0.510-2.019 | 0.967 |
| N2 | 1.636 | 0.795-3.365 | 0.181 |
| N3 | 0.820 | 0.188-3.573 | 0.792 |
| TNM |  |  |  |
| 1 | 1 |  |  |
| 2 | 1.241 | 0.612-2.517 | 0.548 |
| 3 | 1.564 | 0.775-3.157 | 0.212 |
| 4 | 0.869 | 0.190-3.985 | 0.857 |
| NLPR |  |  |  |
| <0.010 | 1 |  |  |
| ≥0.010 | 3.521 | 1.939-6.394 | <0.001* |
| AISI |  |  |  |
| <113.923 | 1 |  |  |
| ≥113.923 | 1.782 | 0.925-3.434 | 0.084 |
| SIRI |  |  |  |
| <1.130 | 1 |  |  |
| ≥1.130 | 2.061 | 1.187-3.578 | 0.010* |
| NMR |  |  |  |
| <7.753 | 1 |  |  |
| ≥7.753 | 2.698 | 1.502-4.848 | 0.001* |
| MSIS |  |  |  |
| 0 | 1 |  |  |
| 1 | 2.012 | 1.032-3.925 | 0.040* |
| 2 | 1.920 | 0.866-4.256 | 0.108 |
| PNI |  |  |  |
| <50.625 | 1 |  |  |
| ≥50.625 | 0.348 | 0.184-0.658 | 0.001* |
| NLR |  |  |  |
| <2.331 | 1 |  |  |
| ≥2.331 | 2.719 | 1.578-4.686 | <0.001* |
| LMR |  |  |  |
| <4.303 | 1 |  |  |
| ≥4.303 | 0.461 | 0.251-0.846 | 0.013* |
| PLR |  |  |  |
| <151.573 | 1 |  |  |
| ≥151.573 | 1.933 | 1.114-3.354 | 0.019* |
| SII |  |  |  |
| <392.944 | 1 |  |  |
| ≥392.944 | 1.691 | 0.968-2.953 | 0.065 |
| WBC | 0.988 | 0.848-1.150 | 0.872 |
| RBC | 0.729 | 0.455-1.168 | 0.189 |
| Hb | 0.991 | 0.976-1.007 | 0.280 |
| PLT | 0.991 | 0.986-0.996 | <0.001* |
| Neut | 1.162 | 0.985-1.369 | 0.075 |
| Lymp | 0.308 | 0.170-0.556 | <0.001* |
| Mono | 1.126 | 0.754-1.681 | 0.562 |
| ALB | 0.944 | 0.877-1.015 | 0.118 |
| Prealb | 0.997 | 0.992-1.003 | 0.356 |
| PT | 1.207 | 0.890-1.637 | 0.227 |
| INR | 0.895 | 0.214-3.741 | 0.880 |
| APTT | 0.996 | 0.926-1.071 | 0.907 |
| TT | 0.945 | 0.844-1.057 | 0.321 |
| FVC | 0.909 | 0.657-1.258 | 0.565 |
| FEV1 | 0.908 | 0.597-1.382 | 0.653 |
| FEV% | 0.999 | 0.985-1.013 | 0.853 |
| DLCO | 0.872 | 0.745-1.022 | 0.090 |
| EF | 1 | 0.882-1.133 | 0.996 |
| Tumor size | 1.131 | 0.953-1.342 | 0.159 |
| Operation time | 1.005 | 1.000-1.010 | 0.033* |
| Intraoperative infusion | 1 | 1.000-1.001 | 0.582 |
|  |  |  |  |
