# Supplementary figures and images for: The predictive value of preoperative inflammatory status for anastomotic leakage after esophagectomy for esophageal cancer
Source: Front Oncol. 2025 Aug 6;15:1587586. doi: 10.3389/fonc.2025.1587586 (PMC12364653; doi:10.3389/fonc.2025.1587586)

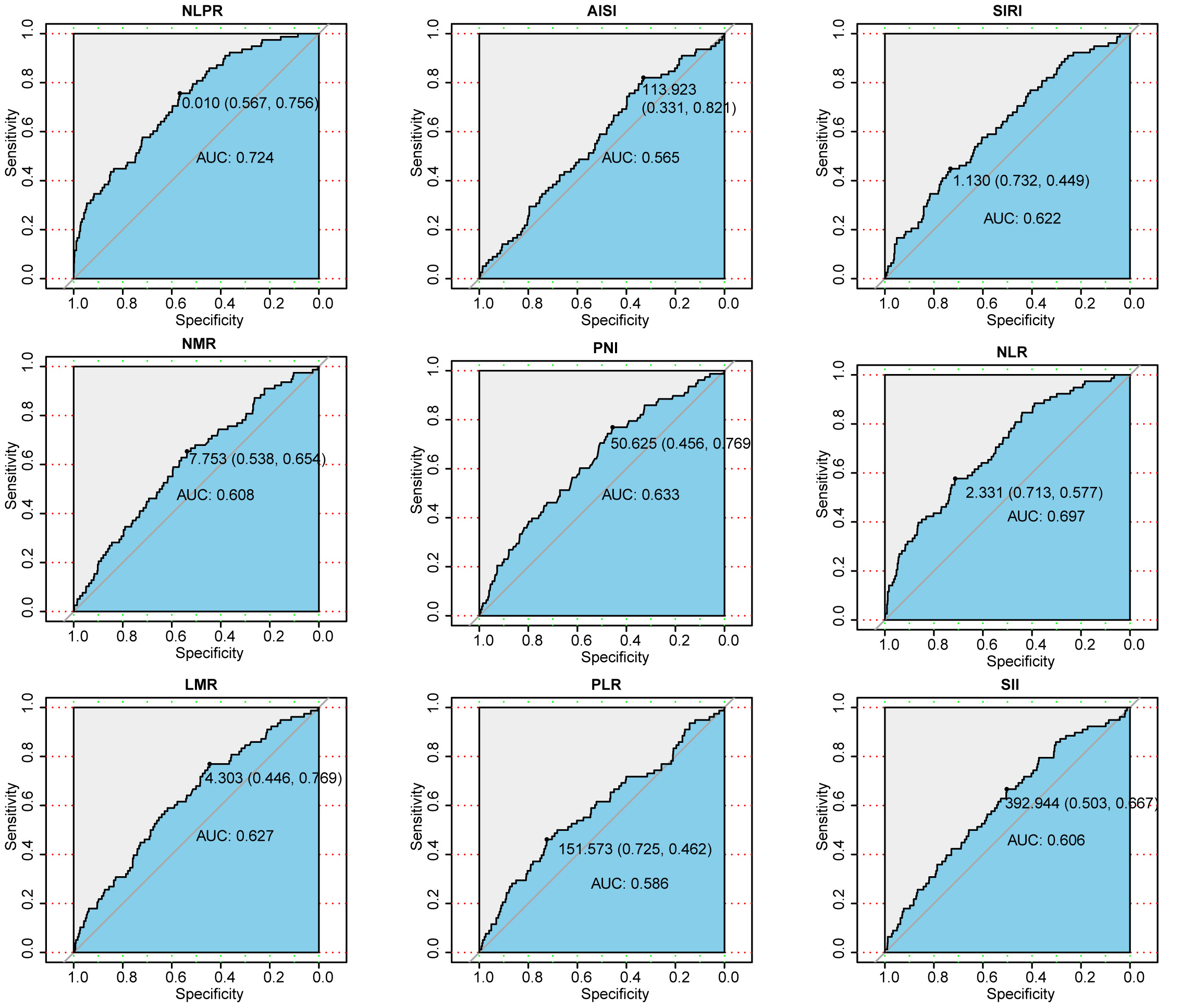

Supplement: Supplementary Figure 1 — Utilizing ROC curve analysis to determine the optimal cutoff values for inflammatory markers. [file Image1.tif]

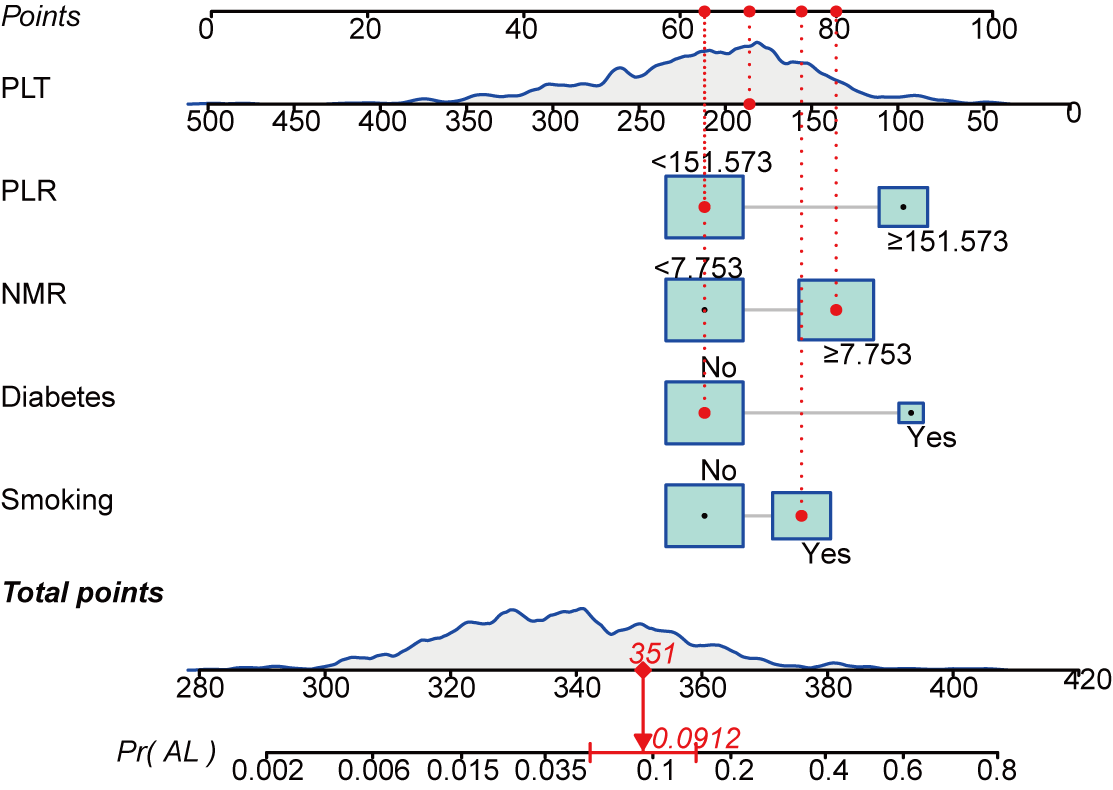

Supplement: Supplementary Figure 2 — A high-resolution nomogram of the training set. [file Image2.tif]
